# Another tick bites the dust: exploring the association of microbial composition with a broad transmission competence of tick vector species

**DOI:** 10.1128/spectrum.02156-23

**Published:** 2023-10-06

**Authors:** Tiago F. Mota, Eduardo R. Fukutani, Kelsilandia A. Martins, Vanessa R. Salgado, Bruno B. Andrade, Deborah B. M. Fraga, Artur T. L. Queiroz

**Affiliations:** 1 Instituto Gonçalo Moniz, Fundação Oswaldo Cruz (FIOCRUZ), Salvador, Bahia, Brazil; 2 Division of Biomedical and Life Sciences, Lancaster University, Lancaster, United Kingdom; 3 Faculdade de Medicina Veterinária da União Metropolitana de Educação e Cultura (UNIME), Lauro de Freitas, Bahia, Brazil; State Key Laboratory of Virology, School of Public Health, Wuhan University, Wuhan, China

**Keywords:** vector competence, tick-borne diseases, microbiome, pathogen transmission

## Abstract

Ticks harbor and transmit many different pathogens. While some species are competent vectors of a single pathogen, mono-competent vectors (MCV), other species are proven to be competent vectors of several pathogens, pluri-competent vectors (PCV). Such a difference in vector competence might be related to the microbiome. To better comprehend its influence on the vector competence of ticks for one or several pathogens, a data-driven approach using publicly available databases was applied on bacterial 16S rRNA from MCV and PCV tick species. Alpha and beta diversity, co-occurrence networks, and functional profiles were analyzed. A differential analysis was performed to identify bacterial genera associated with PCV ticks. These tick species presented higher richness, and the bacterial composition showed a significant difference between MCV and PCV ticks. The bacteria genera of PCV ticks demonstrated fewer correlations within each other in comparison with MCV ticks. The differential analysis revealed 14 bacteria genera related to PCV tick species, such as *Rickettsia*, *Staphylococcus*, and *Corynebacterium*. Using 24 differently abundant genera, tick samples from another data set could be classified into either PCV or MCV with high accuracy and concordance. Moreover, pathway regulation related to reactive oxygen species detoxification, β-Lactam resistance, and dTDP-L-rhamnose biosynthesis could be participating in the competence of PCV ticks for several tick-borne pathogens. These findings enlighten our understanding of the bacteria community’s role on some tick species’ broad vector competence.

**IMPORTANCE:**

Some tick species are competent to transmit more than one pathogen while other species are, until now, known to be competent to transmit only one single or any pathogen. Such a difference in vector competence for one or more pathogens might be related to the microbiome, and understanding what differentiates these two groups of ticks could help us control several diseases aiming at the bacteria groups that contribute to such a broad vector competence. Using 16S rRNA from tick species that could be classified into these groups, genera such as Rickettsia and Staphylococcus seemed to be associated with such a broad vector competence. Our results highlight differences in tick species when they are divided based on the number of pathogens they are competent to transmit. These findings are the first step into understanding the relationship between one single tick species and the pathogens it transmits.

## INTRODUCTION

Vector-borne diseases are responsible for more than 700,000 deaths per year, and around 80% of the world’s population are at risk of infection (1). Among the arthropod vectors, ticks and mosquitoes are responsible for the majority of the vector-borne disease transmission (2). Ticks are obligatory hematophagous arthropods that have a broad host spectrum and are spread worldwide, being an important vector linking pathogens to vertebrates. These characteristics assign them as an important public health problem. Several tick species such as *Ixodes scapularis*, *Ixodes ricinus*, *Haemaphysalis longicornis*, and *Rhipicephalus microplus* are known to be vectors to many pathogens from distinct domains, i.e., virus, bacteria, and protozoa (3). Furthermore, they also impair the society’s economy and public health (4, 5).

Usually, vector-pathogen studies on ticks focus on single-pathogen infections, despite co-infections cases being a commonly noted occurrence on field ticks (6). Although co-infected with many pathogens, these ticks should not be always considered responsible for their transmission. Vector competence represents the arthropod’s ability to acquire a pathogen infection, maintain it with replication, and further transmission to a susceptible host leading to disease development (7). Thus, the incrimination as a vector should be empirically determined to avoid misclassification regarding pathogen transmission (8). Some species, such as *I. scapularis* and *I. ricinus*, were empirically described transmitting bacterial, protozoan, and viral pathogens (9 – 12). On the other hand, other tick species like *Ixodes holocyclus* and *Hyalomma dromedarii* were incriminated as vectors of many pathogens while having little empirical evidence for most of these pathogens. This classification regarding the number of transmitted pathogens was assumed in studies based on molecular detection, which does not prove vector competence (8). However, until now, empirical experiment-based studies suggested that they are competent in transmitting only one single pathogen (13 – 16).

The vector competence of a given tick species to transmit multiple pathogens appears to be related to its microbiome. It was demonstrated that pathogen-microbiome interaction could influence the infection establishment and transmission. The study of tick microbiome enlightened researchers on the tick-pathogen relationship’s complexity, in which symbiont bacteria play an important role in tick fitness and vector competence (17). This type of discovery with the aid of high-throughput approaches enables the development of new control methods focused on the pathogen-microbiome-vector relationship (18). Moreover, a point of view from tick species that are competent to transmit several pathogens in comparison with those that have a narrower vector competence could bring us insights into control mechanisms to decrease such a broad competence. For instance, the introduction of modified symbiont *Sphingomonas* in tick microbiota has been demonstrated to reduce *Anaplasma phagocytophilum* infection in *I. scapularis* ticks (19). Along these lines, the first group of ticks could be assigned as being pluri-competent vectors (PVC) while the second group of ticks as being mono-competent vectors (MCV).

Thus, the aim of the present work was to explore the microbiome data from tick species identified as PCV in comparison with those that are, up to date, considered as MCV. This exploratory approach can help us investigate what tick microbiome can tell us about such a broad vector competence.

## RESULTS

**Fig 1:**
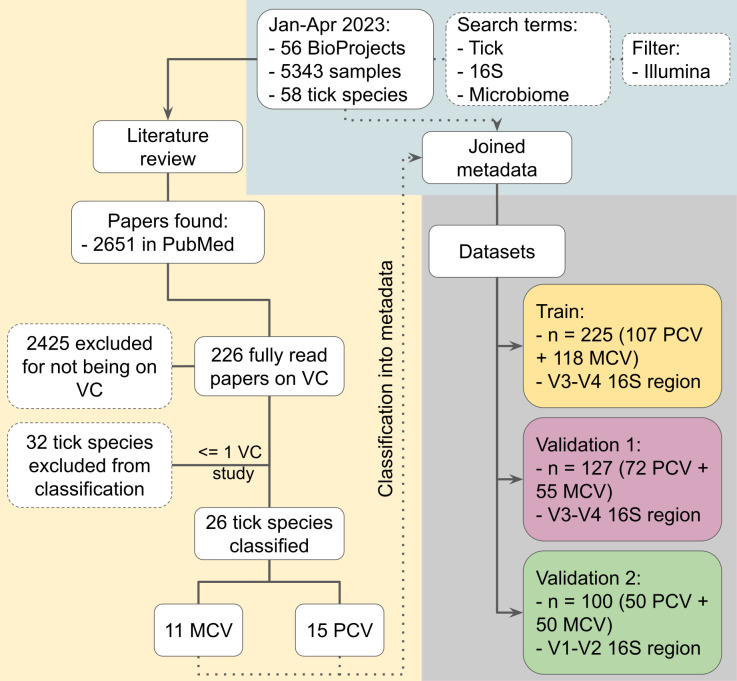
Flowchart of microbiome data search in NCBI’s Sequence Read Archive followed by literature in PubMed review on tick species containing 16S rRNA data. The light-blue area represents the search in the SRA database. The light-yellow area represents the literature review and classification into MCV and PCV tick species. The purple area represents the data sets prepared for the microbiome analysis. VC, vector competence; MCV, mono-competent vector; PCV, pluri-competent vector.

For the literature review on vector competence of ticks that have 16S data deposited in Sequence Read Archive (SRA), 2,651 papers have been screened and 226 empirical vector competence-related studies were fully read, Fig. 1. The majority of these tick species with 16S data in SRA, 32 out of 58, could not be classified into MCV (competent to transmit one or any pathogen) or PCV (competent to transmit more than one pathogen) as only one or no papers have been found that have evaluated its vector competence for any pathogen. Among the 26 species that could be classified based on 226 studies, 17 species had samples with the inclusion criteria for the microbiome analysis herein proposed, i.e., 16S V3-V4 or V4 region, paired-end sequences, 250 to 300 bases, field collected, washed with ethanol, whole body tissue, and free of pathogens. Among the 1,480 samples meeting the inclusion criteria, 352 samples were divided into two data sets. This sample number was selected in order to balance the number of samples of each tick species within each group as some species were overrepresented.

The training data set was used to characterize the microbiota and identify differentially abundant bacteria while the validation data set provided samples to assess the accuracy of the bacteria associated with any of the groups to distinguish them. For the training data set, 225 samples of seven species have been chosen, being the MCV group composed of *Dermacentor silvarum*, *Dasymutilla occidentalis*, and *I. holocyclus* while the PCV group of *Amblyomma cajennense*, *Ha. longicornis*, *I. ricinus*, and *Rhipicephalus sanguineus*. The remaining samples, 127, were used for the validation data set being the MCV group composed of *D. occidentalis*, *D. silvarum*, *Haemaphysalis leporispalustris*, *I. holocyclus*, and *Ixodes persulcatus*. The validation data set’s PCV group was composed of *Dermacentor marginatus*, *Dermacentor reticulatus*, *Dermacentor variabilis*, *Ha. longicornis*, *Haemaphysalis punctata*, *Ixodes pacificus*, *I. ricinus*, and *R. sanguineus*. A second validation data set, 16S V1-V2 region, was composed of 100 samples from four tick species, being *Haemaphysalis humerosa* and *I. holocyclus* assigned to the MCV group while *I. ricinus* and *R. sanguineus* to the PCV group. For each data set, samples were randomly selected in order to balance the number of samples per tick species within each group. Differences of the sample number among groups were not statistically significant when compared with the other data sets by *χ* ^2^ test. The whole classification results and list of tick species and pathogen pairs that have been evaluated can be seen in Table S1.

**Fig 2:**
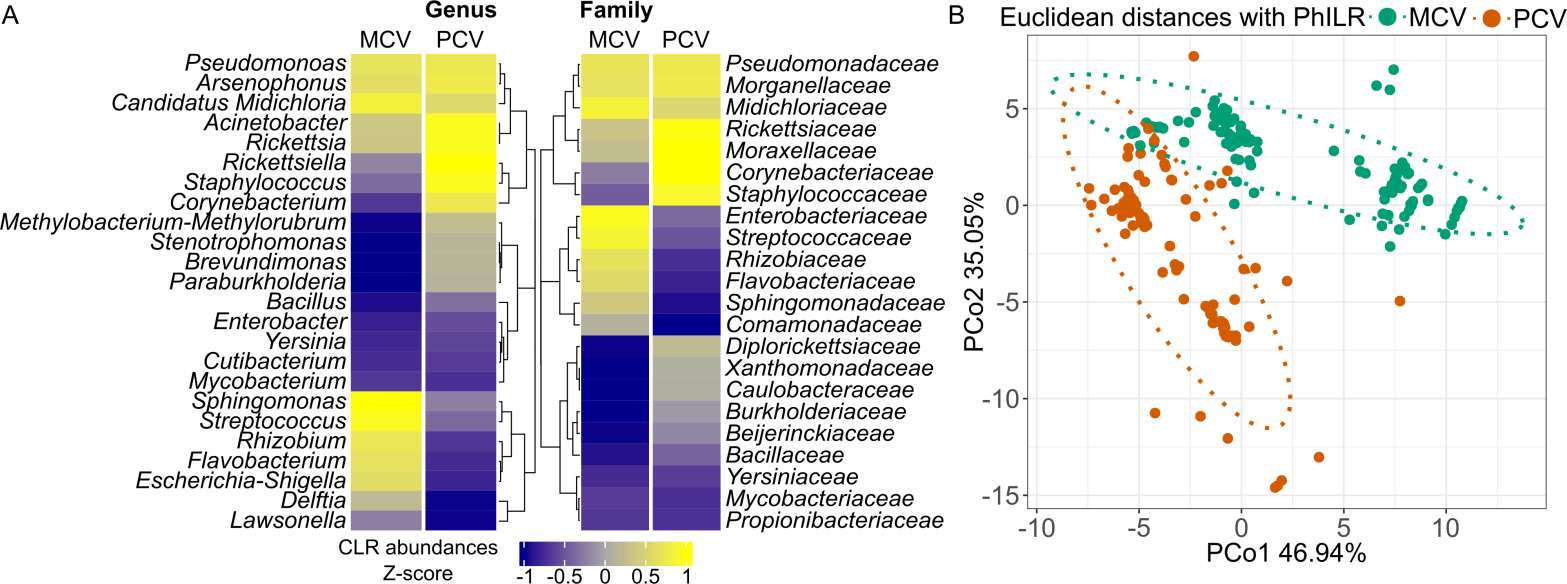
Microbial composition and beta diversity highlighting differences in the bacterial community of mono-competent vectors (MCV) and pluri-competent vectors (PCV). In A, a heatmap using mean CLR transformed abundances by group is represented. Z-score has been applied in order to reduce color scale range. In B, a PCoA from an Euclidean matrix of PhILR transformed counts between groups is represented.

The bacterial community of ticks from each studied group (MCV and PCV) was composed of similar families and genera, however, with different centered log-ratio (CLR) abundances among the groups (Fig. 2A). For instance, *Rickettsiaceae*, *Moraxellaceae*, *Staphylococcaceae*, and *Corynebacteriaceae* families were more abundant in the PCV group. On the other hand, *Enterobacteriaceae*, *Streptococcaceae*, *Rhizobiaceae*, *Flavobacteriaceae*, *Sphingomonadaceae*, and *Comamonadaceae* were more present in the MCV group when comparing both groups in the heatmap, Fig. 2A. In the beta diversity analysis using Phylogenetic Isometric Log-Ratio Transformation (PhILR) followed by an Euclidean distance matrix, MCV and PCV groups presented different bacterial communities as seen in the principal coordinate analysis (PCoA), Fig. 2B. This separation between groups was statistically significant (PERMANOVA, *F* = 93.04, *R* ^2^ = 0.294, *P* < 0.001). According to the PERMANOVA test, 29.4% (*R* ^2^) of the distance between groups could be explained by their vector competence grouping (MCV and PCV). There was no statistically significant difference in multivariate dispersion in the comparison between groups, *F* = 0.18 and *P* > 0.674. Thus, *P-*value inflation in the PERMANOVA test should not be expected.

**Fig 3:**
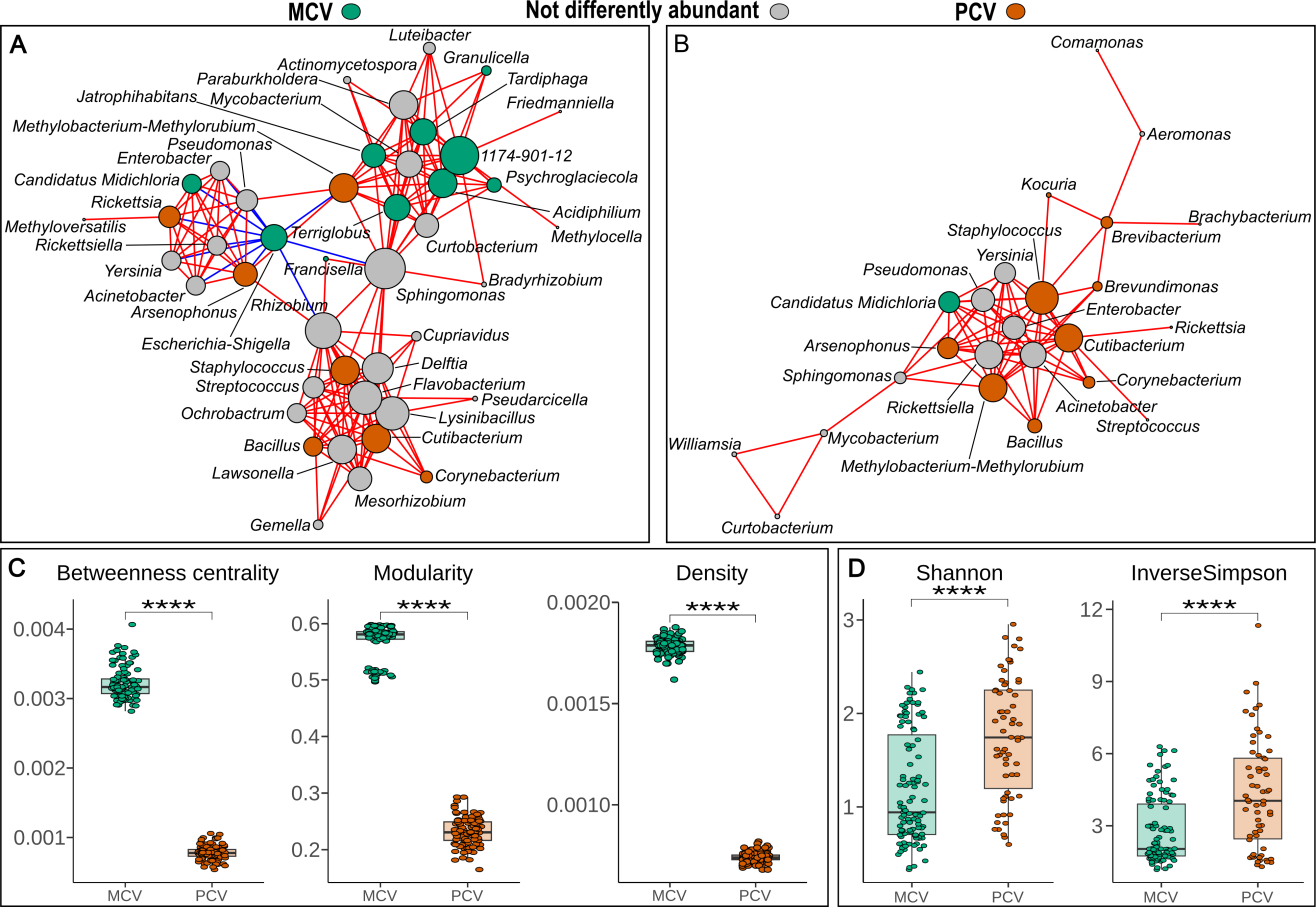
Co-occurrence networks of mono-competent vectors (MCV) (**A**) and pluri-competent vectors (PCV) (**B**) tick species. The node diameter was measured by each node degree. The color of nodes is related to the genera that were differently associated with each of the groups in the linear models for differential abundance analysis. Color of edges represents (red) positive and (blue) negative correlations > 0.4 between genera. In C and D, boxplots of network performance metrics and alpha diversity metrics are shown. Comparisons between groups were done using Mann-Whitney test with *P* < 0.0001.

In order to evaluate how bacteria genera correlated with each other within groups, networks using Sparse Correlations for Compositional data (SparCC) have been built for each group separately. The co-occurrence network of PCV tick species was less modular, with lower overall centrality and density as shown in Fig. 3. Due to this result, the alpha diversity has been evaluated using rarefied counts to see if the smaller and less-dense network seen in PCV tick species was related to a lower diversity of bacteria. Contrasting this previous idea, alpha diversity was higher in PCV ticks as measured by both Shannon and inverse Simpson metrics with Mann-Whitney *P* < 0.0001, Fig. 3D.

Among the 408 pathways predicted with PICRUSt2’s functional analysis, 197 were associated to any of the groups, MCV or PCV, with log_2_ fold change > 1 and BH adjusted *P* < 0.05, according to linear models for differential abundance (LinDA). The pathways predicted to be more active in PCV tick species were related to the biosynthesis of dTDP-L-rhamnose, mycothiol, peptidoglycan, and methyl ketone. On the other hand, the pathways which were predicted to be upregulated in MCV tick species were L-tryptophan biosynthesis, sulfoglycolysis, and degradation of D-galactarate and D-glucarate. A complete table of all differentially regulated pathways can be seen in Table S2.

**Fig 4:**
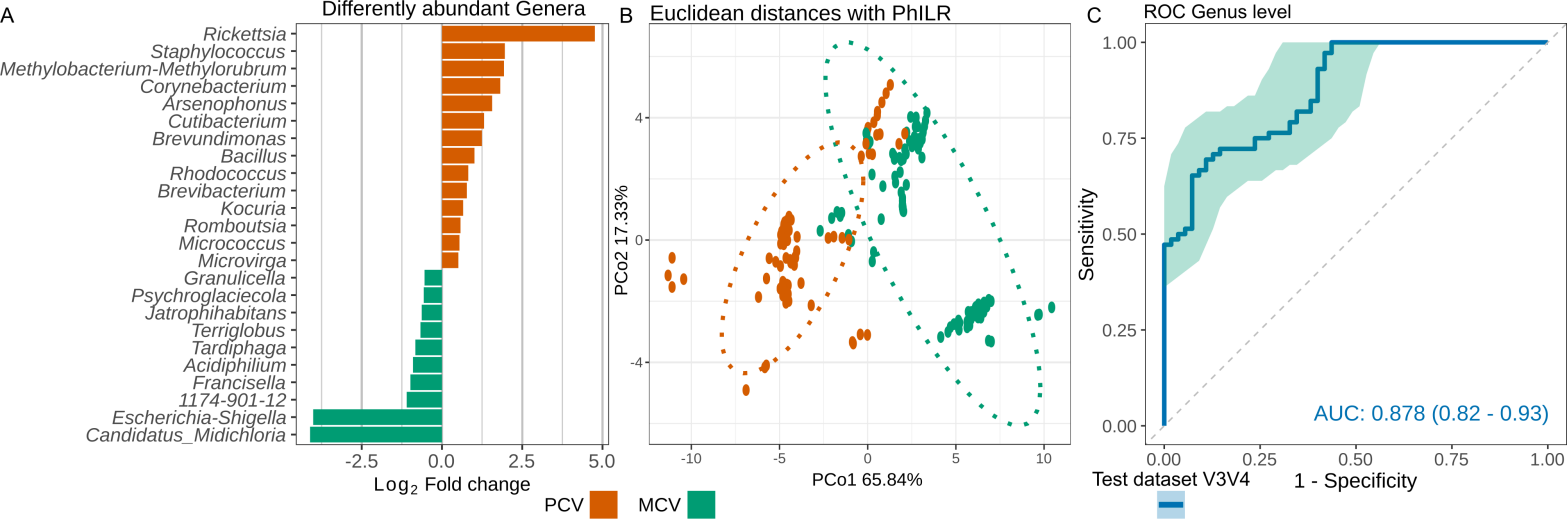
Differential analysis to identify to which group bacteria genera were associated with and validation of using these genera to measure the accuracy of using them to separate samples from another data set into MCV or PCV ticks. In A, LinDA analysis of the microbiota from both mono-competent vectors (MCV) and pluri-competent vectors (PCV) groups. Only taxa with log_2_ fold change > 0.5 and Benjamini-Hochberg adjusted *P* < 0.05 are shown. In B, principal coordinate analysis of the beta diversity, with PhILR and Euclidean distance, when only this group of bacteria was kept. In C, receiver operating curves (ROC) and the area under the curve (AUC) in the train and validation (V3-V4) data sets based on principal component regression.

Several bacteria genera were associated with each of the studied tick groups, presenting LinDA’s log_2_ fold change > 0.5 and BH adjusted *P* < 0.05 (Fig. 4A). Genera such as *Candidatus Midichloria*, *Escherichia-Shigella*, *Francisella*, and *Acidiphilum* were related to the MCV group. To the same degree, *Rickettsia*, *Staphylococcus*, *Methylobacterium-Methylorubrum*, and *Corynebacterium* were associated with the PCV group. In order to validate LinDA analysis results, a PCoA with PhILR and Euclidean distance matrix was used to assess if separation was better using only the genera associated with each group. As seen in Fig. 4B, the clustering separation between the groups was greater than that observed before the filter. In both situations, there was a statistically significant difference in centroid positions from both groups (PERMANOVA, *P* < 0.001), while there was an increased *R* ^2^ value in the abovementioned validation data set from 0.294 to 0.445. In both cases, the homogeneity dispersion of samples within each group showed no statistically significant differences, *F* = 0.18 and *P* > 0.674; *F* = 3.60 and *P* = 0.057, respectively. Using the train data set, i.e., the same data set used for the differential analysis, ticks were classified using a principal component regression followed by a receiver operating curve (ROC) which showed an area under the curve (AUC) of 0.859. When another data set with new samples that sequenced the same 16S variable region, V3-V4, was used, a similar AUC of 0.878 was obtained. Such comparison could not be performed for the second validation data set using samples that have sequenced the 16S V1-V2 region as the 24 differently associated bacteria genera by LinDA analysis were not present in this data set. The model using the 24 genera most associated with MCV or PCV ticks demonstrated a high accuracy of 0.95 (95%CI 0.90–0.98) with a non-informative rate of 0.57 and high concordance, kappa coefficient of 0.90.

## DISCUSSION

The tick species herein evaluated were selected based on the presence of laboratory confirmation and its vector competence being classified as MCV and PCV. Only species assessed under experimental conditions were used. This evaluation was performed by a comprehensive literature review of vector competence studies which highlighted a publication bias in such kind of studies. For instance, some tick species such as *I. ricinus*, *I. scapularis*, and *H. longicornis,* have more studies on their vector competence for any pathogen. These species are most likely to be part of the PCV group as more information regarding their competence to transmit a broader range of pathogens is available in the literature. Regardless of such limitations, several tick species with microbiome data deposited in NCBI’s SRA could be classified as being MCV or PCV allowing us to further evaluate differences in their microbiota.

The alpha diversity results showed a higher richness in PCV tick species. Such observation contrasts what has been seen under empirical condition where *I. scapularis* co-infected with three pathogens, *Borrelia burgdorferi*, *A. phagocytophilum*, and *Babesia microti*, did not show differences in alpha diversity’s Shannon index when compared with pathogen-free ticks (20). Considering our network analysis, the richer PCV group had a network with less correlations among the genera. Such contrast demonstrates that although the microbiota composition of PCV ticks being richer, less correlated microbiota might be important before co-infection for multiple pathogen vector competence, since the samples herein evaluated were free of infection. The microbiota’s beta diversity of MCV and PCV ticks differed in terms of phylogenetic distance as shown in the beta diversity evaluation, PERMANOVA *P* < 0.001, and as multivariate dispersion has not been observed, the *P*-value tends not be over-inflated. Such a difference in beta diversity suggests unique microbial composition related to competence in transmitting several pathogens which was further evaluated with LinDA.

Considering the bacteria genera with LinDA’s log_2_ > 0.5 and BH adjusted *P* < 0.05, the *Francisella* genus was associated with the MCV group, suggesting that bacteria belonging to this genus should direct tick species toward transmitting less pathogens. This genus is composed of pathogenic species as well as endosymbionts that have been identified in many tick species (21). In particular, the latter which are detected in all life stages both in colony-reared and in field-collected ticks (21). High prevalence of *Francisella* was associated with decreased *Rickettsia* genus, which impairs tick borne pathogen transmission by outcompeting other bacterial genera (22, 23). In our analysis, no direct correlation was observed between these two genera.

*Candidatus Midichloria* was also associated with the MCV group. This bacterial genus is closely related to symbionts such as *Midichloria mitochondrii*. This species has been recently demonstrated to be positively associated with *B. burgdorferi* and *Neoehrlichia mikurensis* while being negatively associated with *A. phagocytophilum* (24). However, a method has been used which modeled co-occurrence of bacteria based on the chance of these bacteria being present together with *M. mitochondrii*. As it is a symbiont bacteria present in many ixodid tick species (25), co-occurrence with pathogens does not mean that it plays a role in infection or transmission. In our analysis the genus *Ca. Midichloria* was positively correlated with environmental genera such as *Staphylococcus*, *Enterobacter*, and *Acinetobacter* (26) as well as some symbiont bacteria such as *Arsenophonus sp*. and *Rickettsia sp*. (27). Such correlation with environmental and symbiont bacteria might be indicating a bridge role of *Ca. Midichloria* as environmental bacteria are acquired from habitat and blood meal shaping tick microbiota (26 ). Some of these genera were also associated in the present work with PCV tick species indicating that *Ca. Midichloria* should be important for both evaluated tick groups as it showed positive correlations in both networks.

Recently, *M. mitochondrii*’s pathways and tissue tropism have been studied showing that it may play a role helping the tick by supplying nutrients, increasing its survival as well as maintaining homeostasis and antioxidant defenses (28 ). Thus, this symbiont might be beneficial for the survival of many bacteria that favor correlations of genera. Altogether, it should be promoting an environment associated with both mono- and pluri-competence as suggested by the results of the present work. The other bacteria genera associated with the MCV group have not been related to vectorial competence or any kind of influence on microbial composition yet. Nonetheless, as demonstrated by the results of the present work, they might be somehow affecting bacterial composition leaning vectorial competence for several pathogens.

The *Staphylococcus* and *Corynebacterium* genera are known environmental bacteria that can be acquired by ticks and might interact with tick’s microbiota (26, 29). These genera were associated with uninfected PCV ticks and might, directly or indirectly, favor the colonization of several pathogens in one single tick. The genus *Bacillus* was also associated with PCV ticks and correlated positively with other bacteria related to this same group such as *Staphylococcus* and *Cutibacterium*. In recent work, *Bacillus* has been negatively associated with ticks infected by the pathogen *R. helvetica* while being positively associated with the genus *Rickettsia* (30). In the present work, such a positive correlation with *Rickettsia* was not observed, at least not directly in the PCV network. However, its positive correlation with other genera also related to PCV ticks indicates that it somehow should be helping to promote a favorable environment for the competence to transmit some pathogens.

Although the genus *Rickettsia* has been associated with PCV ticks, it showed more correlations with other genera in the MCV network demonstrating positive correlations with other bacteria related to both groups. Since pathogen-infected samples were removed from the analysis and most of the species belonging to *Rickettsia* are non-pathogenic (31), we believe that this genus detected in our analysis refers to non-pathogenic symbionts. Rickettsial endosymbionts like *Rickettsia peacockii* and *Rickettsia buchneri* have been strongly related to rickettsial pathogens such as *Rickettsia rickettsii* and *Rickettsia monacensis*, respectively (32, 33). However, little is known about the influence of these endosymbionts in tick vector competence (34). Based on the results of the present work, these bacteria should be somehow related to the transmission of several pathogens by one single tick species.

Although geographically distinct populations of a given tick species have microbiomes with different composition, the functional profile is conserved and redundant within tick species (35). The results from the functional analysis showed a higher predicted regulation of mycothiol biosynthesis in PCV ticks which is associated with reactive oxygen species (ROS) detoxification as well as protection against electrophilic compounds (36). Such a defensive mechanism in PCV ticks against ROS and electrophilic compounds might be contributing with the broader vector competence of these tick species as it facilitates pathogen replication and transmission (37).

Biosynthesis of dTDP-L-rhamnose is an important pathway for viability or virulence of many bacterial pathogens such as Group B *Streptococcus*, *Enterococcus faecalis*, and *Pseudomonas spp*. (38). This pathway was associated with PCV ticks, and since it is absent in mammals (39), it might be an interesting aim to modulate vector competence of this tick group in order to make it less competent to transmit many pathogens.

It has been shown that *Rickettsia* and *Corynebacterium* genera were related to penicillin degradation pathways (40). Thus, these genera might be related to the higher β-lactam resistance predicted by PICRUSt2 analysis in PCV ticks, i.e., higher peptidoglycan biosynthesis V β-lactam (MetaCyc). Both of these genera have been associated with PCV ticks and together with the higher β-lactam resistance might represent an important mechanism to promote many pathogen development.

Most of the studies evaluating functional differences in the host-pathogen relationship test one single pathogen versus one single host. Our results alongside these one-to-one host-pathogen interactions from previous studies have shown important shared pathways that contribute with higher β-lactam resistance regulation and tick’s redox balance mechanisms. Altogether, these pathways might be related to the competence to transmit several pathogens.

After selecting the bacterial genera associated with any of the tested groups, beta diversity results differed in terms of *R* ^2^ being about 1.5 times higher after picking taxa with LinDA’s log_2_ > 0.5 and BH adjusted *P* < 0.05. This demonstrates that these bacteria are better able to distinguish MCV and PCV tick groups by PhILR and Euclidean distance. However, this performance should be applicable only to other tick samples that had sequenced the 16S rRNA V3-V4 region as it had high accuracy, 0.95 (95% confidence interval 0.90–0.98) when validated with another set of samples from this same variable region. Validation with V1-V2 data set could not be tested as none of the 24 genera indicated by LinDA analysis were present in this data set. This difference in bacterial composition should be associated with the lower comparability between data sets from different 16S variable regions due to specificities in bacteria classification of each region (41).

Several bacteria groups related to PCV tick species are commonly found in the environment which might be related to the sequenced tick tissue and pre-washing step. Samples herein analyzed had the tick’s whole body sequenced and were surface washed with ethanol. Such a cleaning method is known to be the least effective for surface decontamination when compared with other methods, such as 5% sodium hypochlorite (42). However, if samples that had been previously washed with 5% sodium hypochlorite were chosen as selection criteria for the analysis, most of the tick samples would have been Australian ticks which sequenced the V1-V2 16S rRNA region. This criterion would have decreased the influence of environmental bacteria in the analysis; nevertheless, it would have inserted a geographical bias as well as diminished the number of tick species from both groups.

It is noteworthy that the relationship between environmental bacteria and tick microbiota has been proposed affecting its composition (43, 44). Thus, these bacteria may play a role in the tick microbiota indirectly affecting the tick’s vectorial competence for many pathogens. Silva’s 16S rRNA database presents limitations to accurately identify bacteria to the species level (45). Therefore, the analysis of the present study was limited to the bacteria family and genera level. Such accuracy level bewilders the separation of species that could be actually part of the environment from those that had already established a co-evolutionary relationship in the tick microbiome and thus play a role in the vector competence of PCV tick species. Although environmental bacteria could not be removed from our analysis, assuming the influence of such bacteria in microbial composition of ticks, changes in the environment could help us modulate the tick microbiome (46). Thus, the bacteria genera herein appointed as associated with the MCV group, such as *Escherichia-Shigella* and *Francisella*, could help us, until a certain point, diminish the vectorial competence for several pathogens of a PCV tick species. As an example, such an approach can be performed as recently proposed by Mazuecos and collaborators (19) where modified symbiont *Sphingomonas* promoted a reduction of *A. phagocytophilum* in *I. scapularis* ticks. Thus, changing the tick’s microbiota using the abovementioned bacteria associated with mono-competent vector ticks could be a method to reduce such a broad vector competence. Naturally, due to the abovementioned limitations regarding the samples, predictions, and mechanisms, the results of the present work should be empirically validated in the future for better comprehension of the mechanisms related to PCVs.

### Conclusion

Based on our exploratory approach on the microbiome of uninfected PCV versus MCV ticks, the bacteria genera *Rickettsia*, *Staphylococcus*, and *Corynebacterium* and *Arsenophonus* among others are related to a broader vector competence. Additionally, pathways related to ROS detoxification, dTDP-L-rhamnose biosynthesis, and β-lactam resistance regulation could be participating in the infection of several tick-borne pathogens in ticks. These findings could be used in the future to regulate the permissiveness of PCV ticks in order to reduce their vector competence or even block the transmission of several pathogens by one single tick species. As we used and integrated publicly available data, it was not possible to determine the species level for all amplicon sequence variants (ASVs). Due to this limitation, the genus level was used for the major analysis. Deeper insights would be found evaluating each bacteria species on those ticks. Despite these limitations, we were able to provide understanding on the bacteria community role on the vector competence of tick species studied.

## MATERIALS AND METHODS

### Vector competence literature review

For this analysis, tick species that have 16S rRNA gene sequencing data in the SRA and NCBI’s BioProject, between January and April 2023, were selected. Following, a literature review was performed, assessing studies which evaluated the tick species’ vector competence for one or more pathogens. Thus, all 58 tick species with 16S rRNA microbiota data deposited in SRA were reviewed for their vector competence for pathogens of medical and veterinary importance. Reviews were carried out for each species through systematic searches, using the species name and terms related to vector competence (i.e., “Vectorial competence” OR “Vector competence” OR “Vector capability”) as well as pathogen transmission pathways (vertical OR horizontal OR transstadial OR transovarial AND transmi*). The searches were performed on the NCBI’s PubMed. When less than 15 studies were found, only the species name was used for the search and all result titles and abstracts were filtered to find works that evaluated the vector competence of the species in a laboratory setting.

To assess the vector competence of each species, tick species and pathogen pairs were classified according to the degree of evidence presented, which used adaptations of the criteria previously outlined in the literature (8). Thus, the criteria to determine vector competence were experimental demonstration of the following: (i) acquisition of pathogens when feeding uninfected ticks on experimental infective hosts, (ii) maintenance of pathogens during the seedlings of experimentally infected tick life stages (vertical transmission and/or transstadial passage), and (iii) transmission of pathogens to a susceptible host in a subsequent blood meal.

Each study was evaluated, focused on demonstration of any of these criteria, and a tick/pathogen pair was classified according to the level of evidence presented by the applied method. The criteria were as follows:

- Grade 1 when there was only detection of the pathogen in the tick collected in endemic areas;
- Grade 2 when ticks collected from the field were evaluated in the laboratory for transmission of pathogens to susceptible hosts or to other life stages (vertical or transstadial transmission) as well as laboratory experiments using artificial infections;
- Grade 3 when there was experimental demonstration of at least one of the aforementioned criteria using colony-reared ticks;
- Grade 4 when all vector competence criteria have been demonstrated in the laboratory.

Species were classified as competent to transmit a given pathogen only when there were studies that evaluated vector competence with evidence degrees 3 or 4 and empirically demonstrated such competence. Despite existing works declaring vector competence for certain pathogens, if they were based only on pathogen detection in ticks or on citations of another published work without enough vector competence evidence, the tick species was still classified as an incompetent vector for such pathogens. When tick species presented vector competence for more than one pathogen with a grade 3 or 4 evidence degree, it was classified as being PCV. On the other hand, if it showed vector competence for only one or any pathogen with grade 3 or 4 evidence degree, it was classified as MCV. A table summarizing the findings and classification of the review can be found in Table S1.

### Data selection and tick classification

From the data on each tick sample with bacterial 16S-rRNA sequencing deposited at the NCBI, a metadata was assembled containing information such as life stage, pre-sequencing washing method, variable region of the 16S gene, tick collection location, and tick species. A new variable was added regarding the number of pathogens that each tick species is competent to transmit as the aforementioned classification. In order to reduce the heterogeneity between samples and retain the highest number of tick species, only samples from data sets containing field-collected ticks by flagging, ticks’ whole body, ticks without previous detection of infection of any pathogen, and ticks with prewash using alcohol or alcohol plus some other reagent in the methodology were included. In addition, the sequencing was filtered for V3-V4 or V4 regions with a paired-end layout and 250 or 300 bases as a higher number of samples was attained in comparison with other variable regions.

### Data processing

All samples were downloaded using SRA toolkit’s fastq-dump (github.com/ncbi/sra-tools) and quality checked by Trimmomatic v0.32 (47) to remove the sequencing adapter, small and unspecific reads, and those with Phred quality score lower than 30. After performing the data quality control, all forward and reverse reads have been joined and processed on the QIIME2 2022.11 (48) pipeline, removing chimeric sequences and building ASVs using the Deblur algorithm (49). Then, all ASVs have been classified using the SILVA v138.1 16S database (50) assuming 97% identity. Before usage, the database was filtered to remove sequences that could be associated with fungi, filtering out Eukaryota, plants, removing Mitochondria and Chloroplast, and sequences with unspecified species classification such as *unkown*, *uncultured*, NA, *metagenome*, and *unidentified*. After classification, a phylogenetic tree was built using MAFFT (multiple alignment using fast Fourier transform (51) and fasttree (52) methods. For the analysis, all of these data generated on QIIME2, i.e., ASV table, ASV taxonomic classification, and phylogenetic tree, have been imported in R environment v4.2.3 using Qiime2R package (53). This package creates a phyloseq object that can be further analyzed by the Phyloseq package (54) to measure diversity indices.

### Batch effect correction

Before any analysis, a batch correction has been performed to reduce the technical bias variation that could have been caused by batch, e.g., each project being sequenced and performed by different research groups and sequencing machines. Such correction was performed by the ConQuR_libsize function (55), from the ConQuR package v2.0. After batch correction, the corrected ASV table was incorporated in a phyloseq object. For future analysis, samples with less than 100 reads and taxa with less than 10 counts have been removed.

### Compositional and diversity analysis

To visualize the microbial composition of MCV and PCV tick species, heatmaps using the batch-corrected mean counts transformed by centered log-ratio were created. CLR transformed counts were scaled using Z-scores in order to reduce the range of values for better visualization of differences between groups. As the alpha diversity metric could not be measured using CLR transformed counts, the rarefied batch-corrected counts have been used for such analysis, rarefying for 1,000 counts and using Shannon and Inverse Simpson indices. Beta diversity was assessed by means of ASV count transformation using PhILR (56), followed by a Euclidean distance matrix. Such an approach takes into account the data’s compositionality characteristic together with the phylogenetic distance from taxa present in samples. Principal coordinate analysis was applied to evaluate group distances in terms of beta diversity followed by PERMANOVA test (57), in order to determine statistically significant differences in each group centroid according to the Euclidean distance matrix. The multivariate dispersion has been tested through the vegan’s package v2.6.4 betadisper function to assess the homogeneity of each sample distance in relation to its group centroid. If groups are equally homogeneous, then the *P*-value from the PERMANOVA test is not inflated.

### Co-occurrence network

Co-occurrence networks have been built using Sparse Correlations for Compositional data between samples from each group, MCV and PCV. In order to compare networks from both groups, 100 bootstrap replicates of SparCC correlation matrices were made to extract network performance metrics such as betweenness centrality, modularity, and density. After collecting these metrics from 100 bootstrapped networks, they were compared between MCV and PCV groups with the Mann-Whitney test. The package ggClusterNet v0.1.0 has been used to measure SparCC correlation matrices, and networks were built using igraph v1.4.1.

### Functional analysis

In order to further describe PCV ticks, a functional analysis was performed using PICRUSt2 (Phylogenetic Investigation of Communities by Reconstruction of Unobserved States), predicting metagenome functions that are regulated by a given bacterial community based on 16S-rRNA sequencing (58). Predicted pathways were described according to the MetaCyc database and further hierarchically classified into superclasses as provided by the file2meco R package v0.5.1. Differently abundant pathways were identified using Linear models for Differential Abundance (59) with a cut-off of 1 log_2_ fold change and Benjamini-Hochberg false discovery rate (BH) adjusted *P* < 0.05.

### Differential analysis and validation of differentially abundant bacteria

To determine groups of bacteria with higher probability to explain differences among groups (MCV vs PCV), LinDA has been applied as it takes into account the data’s compositionality. Differently abundant genera related to MCV or PCV were determined when log_2_ fold change was >0.5 with an BH adjusted *P* < 0.05. Tick life stages have been used in the LinDA model to adjust the vector competence variable.

The bacteria identified as differentially abundant between MCV and PCV ticks in LinDA were used in a principal component regression approach to assess if these bacterial genera accurately separate tick species as MCV or PCV. Thus, only bacterial genera with the abovementioned fold change and *P*-value criteria were used to run a PCA. The first two components were modeled in a logistic regression to determine if samples belonged to any of the studied tick groups. A validation was performed on a data set, composed of other tick samples which sequenced V1-V2 and V3-V4 16S rRNA regions and were unused in the differential analysis as well as samples from other tick species in both groups. The performance of the logistic model built from the train data set was measured using the abovementioned validation data sets containing only the differently abundant bacterial genera. Receiver operating characteristic curves, package pROC v1.18.0, and the confusion matrix method, package caret v6.0.94, have been used to measure the accuracy, non-informative rate, and concordance kappa between train and test data sets.

## Data Availability

Raw sequences were obtained from NCBI’s SRA under the following BioProjects: PRJEB36903, PRJNA574713, PRJNA577275, PRJNA631062, PRJNA661974, PRJNA664219, PRJNA732915, PRJNA733831, PRJNA766341, PRJNA801881, PRJNA352452, PRJNA401547, PRJNA438789, PRJNA494526, PRJNA523509, PRJNA530927, PRJNA548395, PRJEB46006, PRJNA640465, and PRJNA559059. Metadata and code are available upon request.

## SUPPLEMENTAL MATERIAL

Supplemental tables spectrum.02156-23-s0001.xlsx Results from the literature review and differential functional analysis.

ASM does not own the copyrights to Supplemental Material that may be linked to, or accessed through, an article. The authors have granted ASM a non-exclusive, world-wide license to publish the Supplemental Material files. Please contact the corresponding author directly for reuse.
